# Adenocarcinoma of the lacrimal gland: a case report

**DOI:** 10.1186/s13256-017-1412-2

**Published:** 2017-09-11

**Authors:** Asmae Touil, Sarah El Abbassi, Yassine Echchikhi, Mustapha Maher, Tayeb Kebdani, Noureddine Benjaafar

**Affiliations:** 1 Department of Radiotherapy, National Institute of Oncology, Rabat, Morocco; 2 Department of Pathology, Specialties Hospital, Mohamed V University, Rabat, Morocco

**Keywords:** Lacrimal gland, Adenocarcinoma

## Abstract

**Background:**

Primary ductal adenocarcinomas of the lacrimal gland are very rare. This neoplasm shares some histological and immunohistochemical similarities with salivary duct carcinoma.

**Case presentation:**

Here, we present a case of a 55-year-old Moroccan man with lacrimal gland adenocarcinoma. He underwent orbital exenteration with lymph nodes dissection and ipsilateral parotidectomy. After surgery, he was lost to follow-up and died 13 months later.

**Conclusions:**

Lacrimal gland tumors are rare but highly aggressive salivary gland tumors. Complete excision with adjuvant radiotherapy is recommended.

## Background

Malignant epithelial tumors of the lacrimal gland are rare and constitute less than 5% of all biopsied orbital lesions [1]. Primary ductal adenocarcinomas of the lacrimal gland are very rare, accounting for only 5 to 7% of epithelial tumors of the lacrimal gland [2]. They are often classified according to the histologic classification of salivary gland tumors because of their morphopathological similarities [1–4]. Primary ductal adenocarcinoma is generally regarded as an aggressive malignancy; however, the best treatment and overall prognosis are unknown [2], which may suggest that primary ductal adenocarcinoma of the lacrimal gland has a poor prognosis [4]. Here, we report a case of adenocarcinoma of the lacrimal gland with a review of the literature.

## Case presentation

**Fig. 1:**
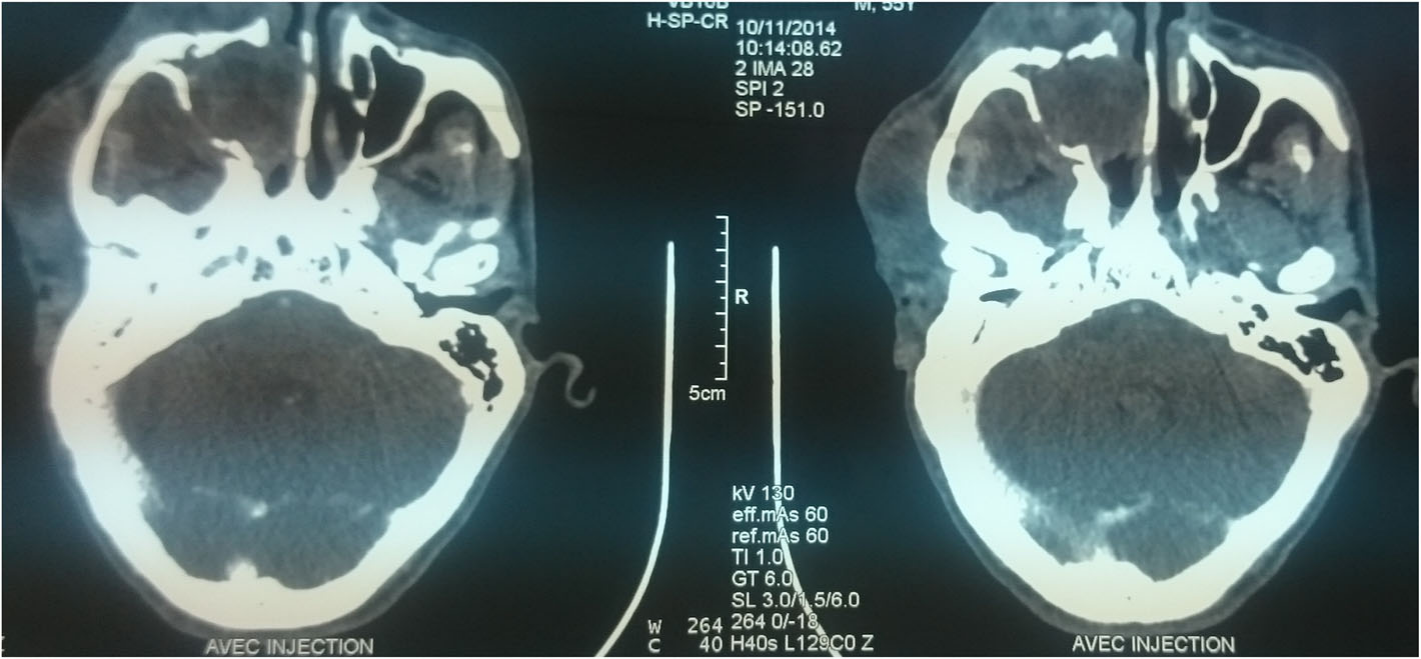
Computed tomography scan shows a mass in the right orbit

**Fig. 2:**
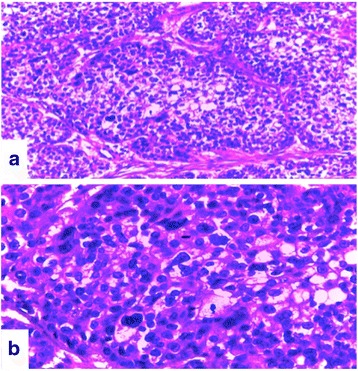
**a** Cancerous tumor proliferation showing a massive arrangement in a fibrous stroma, cell aspect is clarified; Giemsa ×20. **b** Carcinoma cells showing cytonuclear atypia and mitotic activities; Giemsa ×40

Our patient was a 55-year-old Moroccan man with no previous medical history but a traumatic injury on his lower right eyelid 7 years earlier. On the same area, he presented a small ulcerated swelling that had rapidly progressed to his upper and lower right eyelid. The growth was associated with ipsilateral cervical and parotid lymphadenopathy. A computed tomography scan revealed an infiltrative orbital mass measuring approximately 3 to 4 cm (Fig. 1). He underwent an orbital exenteration with lymph nodes dissection (area Ib, II, III, and IV) and ipsilateral parotidectomy. Histological and immunohistochemical studies showed a poorly differentiated adenocarcinoma (Fig. 2). The periocular fat tissue was invaded, his eyeball was intact, the upper and lower palpebral margins and the bone resection margins were adequate, and the periosteum of his right orbit was invaded; of the 27 lymph nodes removed, one was positive. Eight months after surgery, during which he had failed to attend follow-up, he returned with a large recurrence. A computed tomography scan showed a right-sided hemifacial lesion responsible for bone lysis, destruction of his eyeball, and an extension to the maxillary sinus, nasal cavity, and deep spaces of his face. He was fit for chemoradiation but he refused treatment and he died 5 months afterwards.

## Discussion

Tumors of the lacrimal gland constitute less than 5% of all biopsied orbital lesions [3, 4]. The most common epithelial malignancy is primary adenoid cystic carcinoma; adenocarcinoma is rare, representing only 5 to 7% of epithelial tumors of the lacrimal gland [2–6]. Because there is no specific histopathologic classification for lacrimal gland tumors, they are often classified according to the histological classification of salivary gland tumors with which they share many similarities [1, 3, 7].

This subtype of lacrimal gland, adenocarcinoma, was described for the first time in 1996 by Katz *et al.* [2]. Since then, most reports in the literature are case reports describing an aggressive clinical pattern and poor prognosis [1, 2, 8].

The tumor usually begins in the upper eyelid as a growth and is accompanied by clinical symptoms such as lid pseudoptosis, exophthalmos, dystopia, pain, and reduced visual acuity [4, 6].

The patient’s short life expectancy after surgical treatment for adenocarcinoma is due to the early tendency of these tumors for lymphatic invasion and dissemination in the nasal cavity, paranasal sinuses, and more generally in the cranio-orbital region [2, 4].

The tumor is very aggressive [1, 8] with an early affinity for local and distant metastasis and high rates of local recurrences [2, 3]. The death rate of these tumors is approximately 70%, and it usually occurs 2 to 3 years after the initial appearance of the tumor [6]. The most common sites of metastasis are the lung, the bones, the liver, and the brain [2, 6].

The purpose of the treatment is early locoregional control. Complete excision with adjuvant radiotherapy is recommended [3, 4]. Regional lymph node involvement should be methodically evaluated; regional lymph node dissection and/or radiotherapy should be undertaken even in the absence of palpable lymphadenopathy [2].

## Conclusions

It is well known that the lacrimal glands can develop aggressive tumors, primary adenocarcinoma for example. Due to the lack of data on this condition, the clinical behavior, prognosis, and treatment of these tumors are still poorly defined. Early recognition of this highly aggressive tumor ultimately may help to delineate its management.
